# Peonidin-3-*O*-glucoside and Resveratrol Increase the Viability of Cultured Human hFOB Osteoblasts and Alter the Expression of Genes Associated with Apoptosis, Osteoblast Differentiation and Osteoclastogenesis

**DOI:** 10.3390/nu15143233

**Published:** 2023-07-21

**Authors:** Keila C. Ostos Mendoza, Karen D. Garay Buenrostro, Pinal N. Kanabar, Mark Maienschein-Cline, Nina S. Los, Zarema Arbieva, Nishikant A. Raut, Temitope O. Lawal, Alice M. López, Paulina Cabada-Aguirre, Diego A. Luna-Vital, Gail B. Mahady

**Affiliations:** 1 School of Medicine and Health Sciences, Tecnológico de Monterrey, Av. Ignacio Morones Prieto 3000, Sertoma, Monterrey 64710, N.L., Mexico; 2 Clinical Pharmacognosy Laboratory, Department of Pharmacy Practice, College of Pharmacy, PAHO/WHO Collaborating Centre for Traditional Medicine, University of Illinois at Chicago, Chicago, IL 60612, USApaucabada@gmail.com (P.C.-A.); 3 Research Informatics Core, Research Resources Center, University of Illinois at Chicago, Chicago, IL 60612, USAmmaiensc@uic.edu (M.M.-C.); 4 Core Genomics Facility, Research Resource Center, University of Illinois at Chicago, Chicago, IL 60612, USA; nlos2@uic.edu (N.S.L.);; 5 Department of Pharmacy Practice, University of Illinois at Chicago, Chicago, IL 60612, USA; 6 Department of Pharmaceutical Sciences, Rashtrasant Tukadoji Maharaj Nagpur University, Nagpur 440033, India; 7 Department of Pharmaceutical Microbiology, University of Ibadan, Ibadan 200132, Nigeria; 8 Department of Chemistry and Nanotechnology, Tecnológico de Monterrey, Ave Eugenio Garza Sada 2501, Monterrey 64710, N.L., Mexico; 9 Institute for Obesity Research, Tecnologico de Monterrey, Monterrey 64710, N.L., Mexico; dieluna@tec.mx

**Keywords:** apoptosis, Bcl-2, DLX5, HIF1α, IL-18, RNA-seq, *Rankl*, *Sp7/osterix*, osteoporosis

## Abstract

High-throughput RNA-sequencing can determine the impact of nutrients and their combinations on gene transcription levels in osteocytes, and clarify the biological pathways associated with their impact on bone tissues. Previously, we reported that resveratrol (RES) and peonidin-3-*O*-glucoside (POG) increased osteoblastogenesis, as well as reduced osteoclastogenesis in transgenic teleost fish models. Here, we perform whole-genome transcriptomic profiling of osteoblasts treated with POG or RES to provide a comprehensive understanding of alterations in gene expression and the molecular mechanisms involved. Cultured human fetal osteoblastic hFOB 1.19 cells were treated with the test compounds, and then RNA was used to prepare RNA-seq libraries, that were sequenced using a NovaSeq 6000. Treatment with POG or RES increased osteoblast proliferation and reduced apoptosis. Transcriptomic profiling showed that of the 29,762 genes investigated, 3177 were differentially expressed (1481 upregulated, 1696 downregulated, FDR ≤ 0.05) in POG-treated osteoblasts. In the RES-treated osteoblasts, 2288 genes were differentially expressed (DGEs, 1068 upregulated, 1220 downregulated, FDR ≤ 0.05). Ingenuity^®^ Pathway Analysis (IPA) of DGEs from RES or POG-treated osteoblasts revealed significant downregulation of the apoptosis, osteoarthritis and HIF1α canonical pathways, and a significant reduction in Rankl mRNA expression. The data suggest that RES and POG have both anabolic and anticlastogenic effects.

## 1. Introduction

Despite recent advances in treatment, osteoporosis continues to be a serious global health problem, impacting more than 200 million people worldwide and is associated with increased osteoporotic fractures and mortality rates [1,2]. Over the next 20 years, the risk of osteoporosis will continue to increase due to global aging, poor nutrition and a lack of weight-bearing exercise [1,2]. Thus, new integrative and nutritional approaches for the management of osteoporosis, and other metabolic bone disorders are urgently needed. These treatment strategies should focus on new agents that both reduce bone loss and increase bone mass. Despite significant developments of new antiresorptive agents, novel bone-anabolic agents are urgently needed, and should be used in conjunction with prophylactic measures such as optimal nutrition, functional foods, dietary supplements, weight training and fall prevention strategies. 

The maintenance of bone homeostasis is highly organized and controlled by the activities of osteoblasts, osteoclasts and osteocytes, and is regulated by molecular signaling pathways that result in no net loss of bone [3,4,5]. The entire process is essential for maintaining bone mineral density and strength [3,4,5]. When these cells are functioning properly, bone homeostasis is maintained, and net bone mass remains intact. However, during aging and osteoporosis, there is increased osteoblast apoptosis, and the activities of osteoclasts are increased, thereby increasing bone resorption [6]. While new therapies for osteoporosis have primarily concentrated on inhibiting bone resorption, very few drugs have focused on decreasing osteoblast apoptosis to enhance the ability to build bone, in addition to reducing osteoclastogenesis and bone loss.

For more than a decade, research has shown that the ingestion of specific foods containing natural compounds, such as resveratrol and anthocyanins, improves bone mineral density and reduces bone loss [7,8,9,10,11,12,13]. Previously, we reported that anthocyanin-containing extracts of blackcurrants (BCE) and açaí increased osteoblastogenesis and decreased apoptosis in cultured human osteoblasts, and in *Sp7/osterix*:mCherry medaka (*Oryzias latipes*) by increasing *Sp7*/*osterix* and Runx2 expression [10,13]. Furthermore, specific natural compounds from BCE, namely peonidin-3-*O*-glucoside (POG) and cyanidin, significantly increased osteoblastogenesis and reduced osteoclastogenesis in transgenic medaka, while delphinidin-3-*O*-glucoside inhibited Rankl-stimulated osteoclastogenesis in *col10a1*:nlGFP/*rankl*:HSE:CFP medaka [11,12]. Both POG and RES increased *Runx2* and *Sp7/osterix* expression in osteoblasts in double transgenic *Sp7/osterix*:mCherry medaka [11]. In *col10a1*:nlGFP/*rankl*:HSE:CFP medaka, both POG and RES suppressed Rankl-stimulated osteoclastogenesis and bone loss by reducing *Rankl* expression, suggesting that these compounds may not only reduce bone loss but also increase bone mass, and therefore may be useful for the management of metabolic bone disorders, including osteoporosis [11,12]. 

For this investigation, we have performed mRNA-seq using RNA isolated from cultured serum-starved osteoblasts treated with 10% FBS, POG or RES to analyze the effects of these compounds on the transcriptome. Using Ingenuity^®^ Pathway Analyses (IPA), we have correlated differential gene expression induced by these treatments with associated canonical signaling pathways and networks in the osteoblast transcriptome that are involved with their mechanisms of action. Both POG and RES significantly altered differential gene expression (DGE), which overlapped with multiple canonical pathways in cultured hFOB human osteoblasts, including apoptosis, osteoarthritis and hypoxia-inducible factor signaling.

## 2. Materials and Methods

### 2.1. Culture and Treatment of hFOB Osteoblasts

The human fetal immortalized osteoblast line (hFOB 1.19) was obtained from the American Type Cell Culture (Manassas, VA, USA) and grown and maintained in a 1:1 mixture of Ham’s F12 Medium and Dulbecco’s Modified Eagle’s Medium, as we have described [10]. To serum starve the osteoblasts, they were subcultured in a medium that did not contain fetal bovine serum (FBS) for 24 h before treatment. The CellTiter-Glo^®^ Luminescent cell viability assay and Caspase-Glo^®^ 3/7 and Caspase-Glo^®^ 8 apoptosis assays were used, as we have previously described, according to the manufacturer’s protocols [11]. For apoptosis, hFOB cells (serum-starved) and positive controls (grown with 10% FBS) were separately seeded in triplicate in 100 µL/well in opaque-walled 96-well plates at a density of 5 × 10^4^ cells and incubated overnight. POG and RES (1 g/mL) were added to a portion of the wells. Caspase-Glo^®^ 3/7 and 8 reagents (Promega Corporation, Madison, WI, USA) were added and mixed and luminescence was determined using a Synergy HT Plate reader (Biotek, Winooski, VT, USA) and Gen5 1.11 software. 

Purified peonidin-3-*O*-glucoside (POG) and resveratrol (RES) (purity > 95%; Polyphenols, Sandes, Norway) were analyzed as we have described [11]. For treatment, hFOB osteoblasts were plated in opaque-walled 96-well plates (5 × 10^4^ osteoblasts/well) in media containing FBS (10%) or media without FBS and incubated for 24 h. After incubation, RES or POG (1 µg/mL in triplicate) was added per well to serum-starved osteoblasts grown in media without FBS. Positive controls were osteoblasts grown in media containing 10% FBS plus vehicle solvent (0.01% DMSO), while negative controls were cells grown in media without FBS plus 0.01% DMSO. RNA was isolated from harvested osteoblasts using Trizol (ThermoFisher Scientific, Waltham, MA, USA) and used for RNA-Seq. Total RNA was quantified using a NanoDrop™ One Spectrophotometer, and the RNA quality was assessed on an Agilent 4200 TapeStation and RNA Screen Tape as we have previously described [14,15]. Remaining DNA concentrations were <10%.

### 2.2. mRNAseq Library Preparation, Validation, Quantification and qPCR

A Universal Plus mRNASeq kit was used to generate the mRNA-seq library (Tecan, Männedorf, Switzerland, PN: 0520B-A01) with 250 ng of the purified RNA and 15 PCR cycles, as we have described [14,15]. An Agencourt RNAClean XP system was used to clean up the amplified libraries (Beckman Coulter, Brea, CA, USA). Electrophoresis was performed using a 2200 TapeStation system and D1000 ScreenTape (Agilent, Santa Clara, CA, USA) to verify the library fragment size distribution, which was determined to be between 264 and 294 nt. The final library concentrations were determined by PCR analysis. A NovaSeq 6000 was used for sequencing using methods we have previously described [14,15]. For confirmation of gene expression, qPCR was performed as we have described using primers (Origene, Rockville, MD, USA) as listed in Supplemental Figure S3. Briefly, a Power SYBR Green RNA-to-CT 1-step kit (Applied Biosystems, Foster City, CA, USA) was used to reverse transcribe and amplify the total RNA according to the manufacturer’s instructions on a Step One Plus Real-Time PCR System (Applied Biosystem, Foster City, CA, USA).

### 2.3. Bioinformatics, Statistics and Database Annotation

Bioinformatics, statistics and raw data analysis were performed by the Research Informatics Core at the University of Illinois at Chicago (UIC-CRI). FastQC was used to generate the quality-control metrics for the RNA-seq data. Alignment of raw reads with the Human Reference Genome hg38 was performed using STAR and BWA MEM [14,16,17]. The ENSEMBL database (www.ensembl.org, accessed on 7 June 2023) was used to analyze differential gene expression (DGEs). Quantification of ENSEMBL genes was performed with FeatureCounts [18,19]. The exactTest in EdgeR was employed to determine differential expression statistics on raw expression counts [20,21]. Using the correction of Benjamini and Hochberg, *p*-values were corrected for multiple testing with the false discovery rate (FDR; q value) [22]. Categorization of differential gene expression (DGEs) into functional clusters was performed in EdgeR using the gene ontology (GO) function.

### 2.4. Ingenuity^®^ Pathway Analysis (IPA)

Canonical signaling pathways and other biological networks that overlapped with differential gene expression were analyzed using the predicted protein function of ENSEMBL using the Ingenuity^®^ Pathway Analysis software 01-22-01 (Qiagen, Germantown, MD, USA), as we have described [14,15,23]. Alteration of gene expression was filtered by FC of ≤−1 and ≥1 and an FDR ≤ 0.05 to determine overlapping canonical pathways in IPA’s databases [23]. 

### 2.5. Data Availability and Gene Expression Omnibus (GEO) Deposition

Data from the mRNA-seq were deposited into the NCBI’s Gene Expression Omnibus (GEO) [24,25] and are publicly available in GEO, series accession number GSE200684 (https://www.ncbi.nlm.nih.gov/geo/query/acc.cgi?acc=GSE200684, accessed on 7 June 2023).

## 3. Results

### 3.1. Serum Starvation Reduces Growth and Induces Apoptosis in Cultured hFOB Osteoblasts

**Figure 1:**
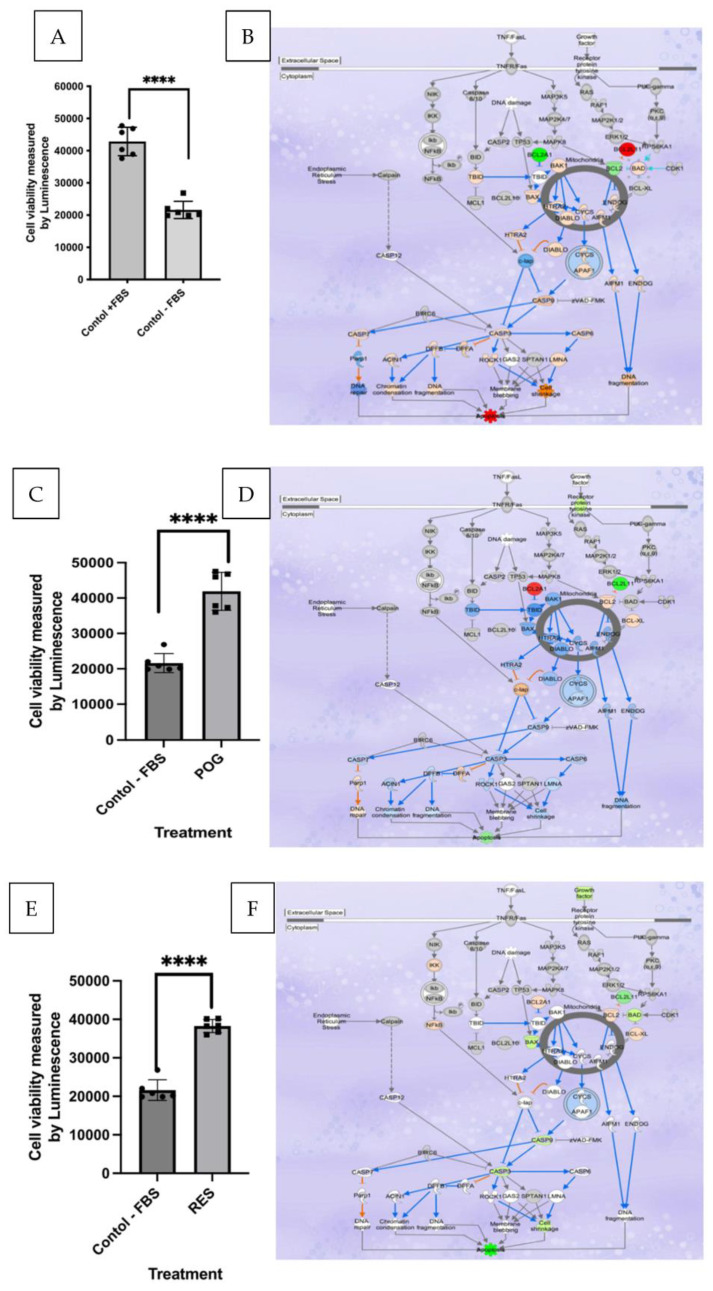
(**A**). Fetal bovine serum starvation of human hFOC osteoblasts (negative control, grown in media without FBS) significantly (*p* < 0.01) reduced osteoblast viability as compared with hFOB osteoblasts grown in media containing 10% FBS (positive control, grown in media containing 10% FBS); n = 6, squares and circles represent data points. (**B**). IPA analysis of the effects of serum starvation (negative controls) as compared with positive control osteoblasts on differential gene expression (DGE) in the apoptosis canonical pathways. (**C**). POG (1 μg/mL) treatment of negative control osteoblasts increased cell viability, measured using the CellTiter-Glo 2.0 assay. (**D**). IPA analysis of DGE showed reduced apoptosis canonical pathway signaling after treatment with POG. (**E**). RES (1 μg/mL) treatment of negative control osteoblasts increased cell viability and reduced apoptosis. (**F**). IPA analysis of the effects of serum starvation (negative controls) versus osteoblasts grown in media containing RES on DGE in the apoptosis canonical pathway. DGE data were analyzed by Ingenuity^®^ Pathways Analysis (IPA), with analysis criteria of FC of <−1 and >1 and FDR < 0.05, using Fisher’s exact test (*p* < 0.05) to determine a significant correlation of canonical pathways with DGEs. Red/pink colors represent significant gene upregulation, and blue/green colors represent significant downregulation of genes. The ENSEMBL database was used to determine DGE enrichment in specific canonical pathways. Osteoblast viability was measured using the CellTiter-Glo^®^ assay, and apoptosis activity was measured using the ApoTox-Glo™ triplex assay. Statistical analysis was performed using GraphPad/Prism 10.0 using the student *T*-test, **** *p* < 0.0001.

The growth of human hFOB osteoblasts subcultured onto media containing no FBS + 0.01% DMSO (serum starvation, negative controls) for 24 h was significantly reduced by >50% (*p* < 0.01) as compared with positive control osteoblasts (media containing 10% FBS + 0.01% DMSO; Figure 1A,B). ATP levels in the negative controls (serum-starved osteoblasts) were also reduced by ~50% and caspase 3/7 activity was increased by 5-fold, indicating apoptosis. Similar to the positive controls (osteoblasts grown in FBS + 0.01% DMSO), treatment with 1 μg/mL of POG or RES increased viability, proliferation and reduced apoptosis in serum-starved osteoblasts as compared with negative controls (Figure 1C–F). Apoptosis was measured by a reduction in caspase 3/7 activities and gene expression in the apoptosis canonical pathway. The ATP concentrations were also increased in serum-starved cells by treatment with RES and POG as compared with negative controls (Supplemental Figure S1).

### 3.2. Transcriptomic and IPA Analysis of Osteoblast Apoptosis

Whole genome transcriptomic profiling showed that of the 29,762 genes that were investigated, positive controls (osteoblasts grown in media containing FBS + 0.01% DMSO) had 5285 differentially expressed genes, with 2861 upregulated and 2424 downregulated (FDR < 0.05) as compared with negative control osteoblasts grown without FBS + 0.01% DMSO. Transcriptomic and IPA analyses of negative control osteoblasts showed that, as compared with the positive controls, negative control osteoblasts had a significantly higher expression of genes involved in apoptosis. They also exhibited a significant downregulation of the antiapoptotic genes, *Bcl-2*, *Bcl-XL* and *CCND1* (Figure 1B). While *BIM* (*Bcl2L11* gene), *BAD* and caspases 3, 6, 7 and 9 were all significantly upregulated (Figure 1B), indicating induction of apoptosis, and decreased cell cycle progression and survival of osteoblasts grown in media without FBS. In addition, the expression of the tumor suppressors p63 and p53 mRNAs was downregulated in the positive control osteoblasts as compared with the negative control treated osteoblasts. 

Analyses of DGEs in IPA showed that, as compared with the serum-starved negative controls, osteoblasts grown in media containing POG (1 μg/mL) or RES (1 μg/mL) exhibited a significantly higher expression of *Bcl-2* genes (Figure 1D,F). For POG-treated osteoblasts, *Bcl-2*, *Bcl-2A1* and *Bcl-XL* were significantly upregulated (FDR < 0.05), while antiapoptosis genes such as *BIM* (*Bcl2L11* gene) were significantly downregulated (FDR < 0.05), favoring a reduction in apoptosis and the survival of osteoblasts (Figure 1D,F). In addition, the tumor suppressor p63 was significantly downregulated (−4 fold, FDR < 0.001). In RES-treated serum-starved osteoblasts, *Bcl-2*, *Bcl-XL* and *Bcl-2A1* were significantly (FDR < 0.05) upregulated, while *BIM* (the *Bcl2L11* gene), *Bax, Bad* and caspases 3 and 9 were significantly (FDR < 0.05) downregulated, favoring a reduction in apoptosis and the survival of osteoblasts (Figure 1F). 

### 3.3. Transcriptomic Analysis of Treated Human hFOB Osteoblasts

**Figure 2:**
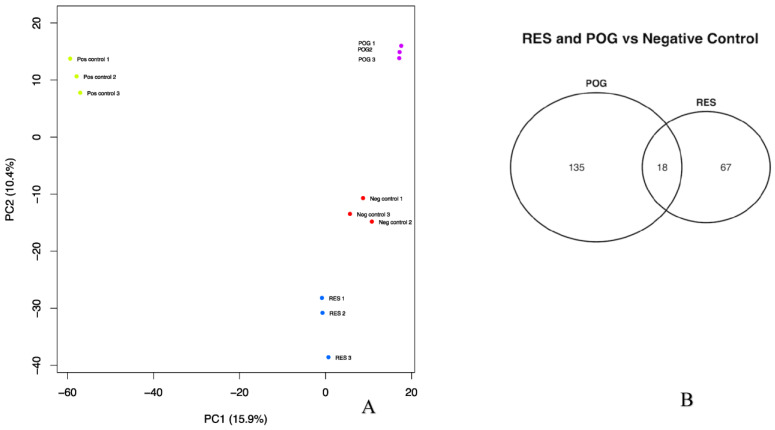
(**A**). Principal component analysis (PCA) of the mRNA-seq results showed differentially expressed genes from serum-starved human hFOB osteoblasts: positive control (+FBS, 0.01% DMSO); negative control (−FBS, 0.01% DMSO) as compared with osteoblasts grown without FBS (−FBS) and treated with RES or POG (1.0 μg/mL). Negative control (red dots; −FBS) samples and positive control (green dots; +FBS); RES-treated samples (blue dots); and POG-treated osteoblasts (purple dots). Differential gene expression was significant for q-values of ≤0.05 (FDR). (**B**). The number of differentially expressed and overlapping genes in RES and POG-treated osteoblasts (FDR < 0.01).

The data from mRNA-seq was used to perform principal component analysis (PCA; Figure 2A). Distinct variations were noted between the positive control osteoblasts as compared with the negative control, as well as the treated (POG or RES, -FBS) hFOB osteoblasts (Figure 2A). The positive and negative controls, and the RES and POG treatment groups, were distinctly clustered and separated from each other in the same PCA plot, validating the different gene expression between positive and negative controls and negative control versus POG or RES treatment groups. An Analysis of 29,762 genes was performed. As compared with negative controls, POG treatment of serum-starved osteoblasts differentially altered the expression of 3177 genes (1481 upregulated and 1696 downregulated, FDR ≤ 0.05). RES treatment of serum-starved osteoblasts showed significant alteration of 2288 genes (1068 upregulated and 1220 downregulated, FDR ≤ 0.05). When compared with negative control osteoblasts, POG and RES had an overlap of only 18 DGEs with a Log FC of <−1 to >1 (Figure 2B). 

Ingenuity^®^ Pathway Analysis (IPA) was used to assess the overlap in DGE after treatment and determine the canonical pathways and molecular networks impacted by treatments. The ratio of DGEs from the mRNA-seq data was correlated with the total number of reference genes in the IPA canonical pathways, using the Fisher’s exact test (*p* < 0.05) to measure significance. The ENSEMBL database was used to determine DEG enrichment in a particular canonical pathway.

**Figure 3:**
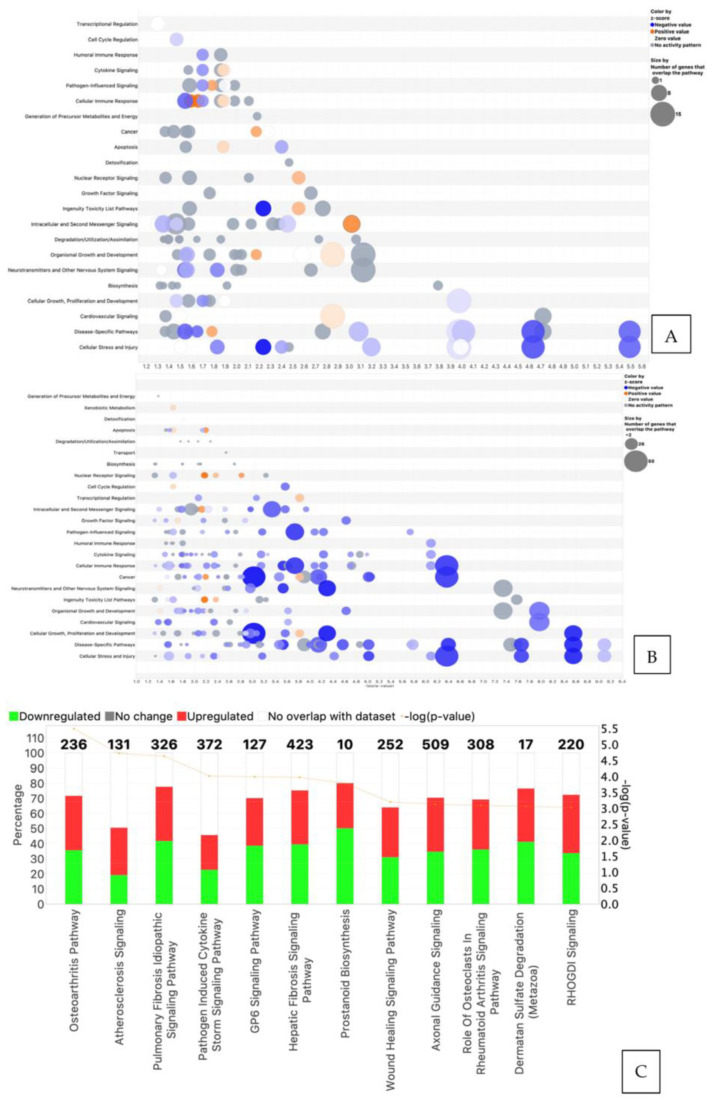

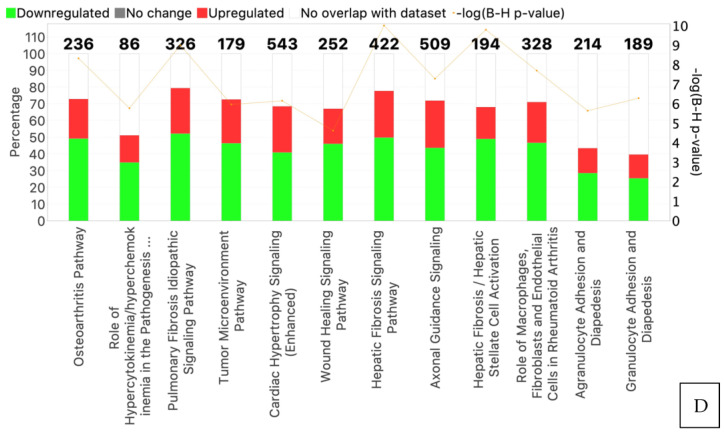
(**A**) Bubble plot of the interconnected canonical pathways associated with differential gene expression in RES-treated serum-starved osteoblasts. Canonical pathway names (*Y* axis) and correlated Z scores (*X* axis), with red bubbles indicating upregulation of the pathway and blue bubbles representing downregulation of the pathway. The bubble size is indicative of the number of genes that overlap the pathway. (**B**) Bubble plot of the overlapping canonical pathways associated with differential gene expression in serum-starved osteoblasts treated with POG. The red/orange colors represent upregulated canonical pathways based on Z-scores, while blue colors represent downregulated pathways. The gray shades are pathways with neutral Z-scores. (**C**,**D**) The most significantly impacted canonical pathways in hFOB osteoblasts treated with RES (**C**) or POG (**D**), *p*-values, % overlap of DGEs and the number of genes (red = upregulated, green = downregulated, Z score > 2.5). Fisher’s exact test (*p* ≤ 0.05) was used to assess the canonical pathways correlated with the DGEs.

IPA analysis revealed that, as compared with negative controls, differential gene expression in POG-treated osteoblasts overlapped with 125 canonical pathways, while DGE in RES-treated osteoblasts overlapped with 53 canonical pathways (Log FC of <−1 to >1, FDR < 0.05; Figure 3A,B). The top 12 canonical pathways (Z-score > 2.5) are shown in Figure 3C,D. The osteoarthritis canonical pathway was the most significantly downregulated pathway (Z-score > 2.5, q < 0.05). A bubble plot with connected canonical pathways impacted by RES treatments of serum-starved hFOB human osteoblasts is presented in Figure 3A. The bubble plot shows the canonical pathway name (*Y* axis) and correlated Z score (*X* axis), with red bubbles indicating upregulation of the pathway and blue bubbles representing downregulation of the pathway. The bubble size is indicative of the number of genes that overlap the pathway. Figure 3B shows the top 12 canonical pathways in RES-treated osteoblasts, with the genes impacted, and their *p*-values, adjusted by the correction of Benjamini and Hochberg for multiple testing (B-H-*p* values). Supplemental Figure S3C is a bubble plot of the interconnected canonical pathways impacted after POG treatment of serum-starved hFOB human osteoblasts. Figure 3D shows the top 12 canonical pathways associated with RES treatment of serum-starved osteoblasts, including the number of genes, and *p*-values, adjusted using the correction of Benjamini and Hochberg for multiple testing (FDR).

**Figure 4:**
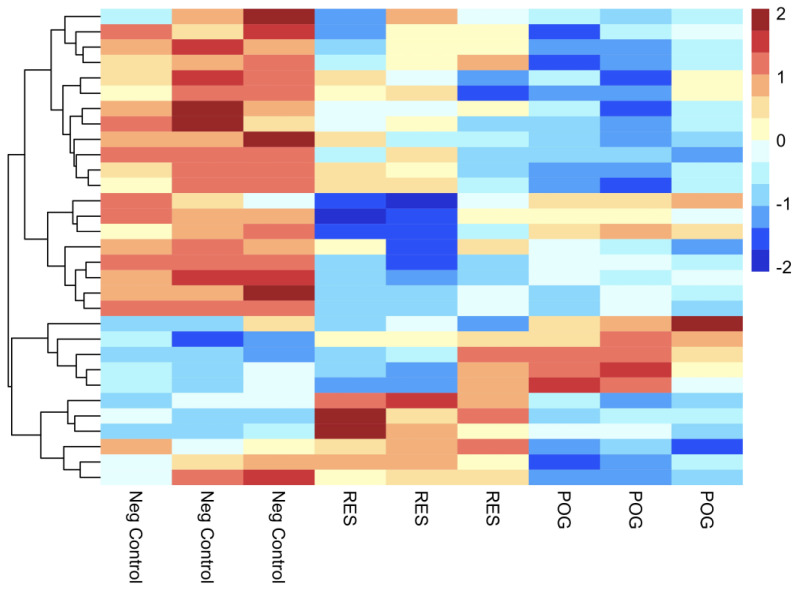
Heatmap visualization of the 30 most significant up- and downregulated DGEs, including gene ontology. Three replicates for negative control hFOB osteoblasts, three for RES treatment and three for POG-treated osteoblasts. The red/pink colors are upregulated genes, and the blue/dark blue colors represent downregulated genes using a Z-scored Log_2_ CPM. A gene ontology enrichment analysis is displayed to show functional distribution.

Figure 4 represents a heatmap of the top 30 DGEs, including hierarchical clustering of DGEs for cultured hFOB osteoblasts treated with POG or RES, as compared with negative control osteoblasts (-FBS + 0.01% DMSO), using a false discovery rate of ≤0.05 and FC of ≤−1 or ≥ 1. Dissimilar gene expression was observed between negative controls and RES- or POG-treated osteoblasts in the heatmap, showing changes in gene expression after treatments and the reliability of the DEGs. The top 10 DGEs are shown in Table 1 for POG-treated and RES-treated serum-starved osteoblasts, respectively. 

### 3.4. Analysis of Osteoblastogenesis, Rankl and HIF Signaling Networks

**Figure 5:**
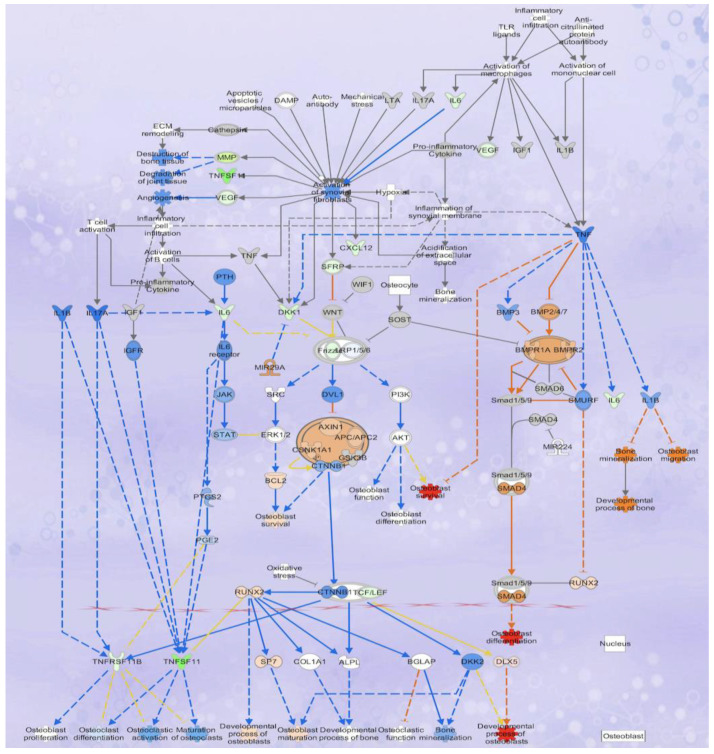
Effects of POG treatment of serum-starved osteoblasts on differential gene expression show upregulation of genes correlated with osteoblast viability and activity, and predicted downregulation of osteoclast differentiation and function. POG treatment significantly (FDR < 0.05) reduced *Rankl* (*THFSF11*) and *IL6* mRNA expression and upregulated the expression of *DLX5*, *Runx2* and *Sp7*/*osterix*, indicating the potential mechanisms by which POG increases osteoblastogensis and reduces osteoclastogenesis. Genes/functions in red/pink depict significant upregulation, while genes in green/light green depict significant downregulation. Genes highlighted in blue represent predicted downregulations of gene expression or function. Genes highlighted in orange show predicted upregulation of gene expression or function.

**Figure 6:**
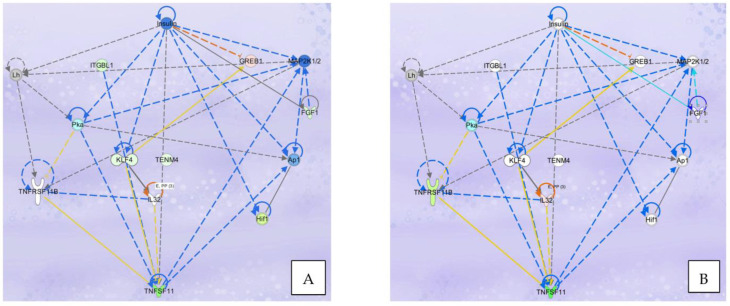
(**A**). Rankl (*TNFSF11*) signaling network effects of POG treatment of serum-starved osteoblasts on differential gene expression. (**B**). Rankl (*TNFSF11*) signaling network effects of RES treatment of serum-starved osteoblasts on differential gene expression. Genes depicted in red/pink show significant upregulation and genes depicted in green/light green show significant downregulation. Genes highlighted in blue represent predicted downregulation of gene expression or function. Genes highlighted in orange represent predicted upregulation of gene expression or function.

The overlap of DGEs in POG and RES-treated osteoblasts with specific canonical pathways and biological networks in the IPA database was analyzed. The primary canonical pathway downregulated was the osteoarthritis signaling pathway (Figure 3C,D) for both POG and RES treatments. In this canonical pathway, POG treatment of serum-starved osteoblasts significantly increased gene expression of *Runx2*, *Sp7/osterix* and *DLX5* (5-fold, FDR < 0.05, Figure 5) as compared with negative controls (Figure 5), while RES-treated osteoblasts showed only a significant upregulation of *IL18* mRNA expression (3-fold, FDR < 0.001). Both positive controls (10% FBS) and POG-treated serum-starved osteoblasts exhibited a significant downregulation of *IL6* mRNA (FDR < 0.01, Figure 5). Both RES- and POG-treated serum-starved osteoblasts exhibited a significant reduction in *Rankl* (*TNFSF11* gene, ~6 fold, FDR < 0.05, Figure 6A,B), as well as *MMP1*, *MMP3* and *ADAM12* mRNA expression (~2–3-fold reduction, FDR < 0.05). qPCR confirmed the mRNA-seq data for POG upregulation of *Dlx5*, *Sp7/osterix* and *Runx2* (Supplemental Figure S3A–C). qPCR analysis of RES- and POG-treated osteoblasts confirmed downregulation of *Rankl* mRNA in treated osteoblasts (Supplemental Figure S3D,E).

Interestingly, POG treatment of serum-starved osteoblasts also led to a reduction in hypoxia induction factor 1α (HIF1α, ~1.5-fold FDR < 0.05, Figure 6A) mRNA expression and canonical pathway signaling. RES or POG treatment of osteoblasts with RES or POG led to a significant downregulation of the long noncoding RNA (lncRNA) *HIF1α-AS2* gene expression (6-fold, FDR < 0.001 for both). In addition, both POG and RES treatments induced a significant increase in *HIF1AN* (an HIF1α inhibitor, FDR < 0.01) mRNA expression, a known HIF1α inhibitor. Hypoxic environments have been shown to reduce osteoblast viability and differentiation [26]. For example, osteoblast activity is lowered in environments with reduced oxygen, and osteoblasts grown in 2% oxygen reduced bone formation ~10 fold [26]. In vivo, osteoblast differentiation was reduced in hypoxic rats [27]. These results suggest that similar positive controls, both POG and RES, may improve osteoblast viability and activity by reducing the hypoxia-inducible response signaling canonical pathway.

## 4. Discussion

Experimental and clinical studies have shown that nutritional and dietary compounds, including anthocyanins, flavonoids, sulforaphane, and resveratrol, reduce bone loss and enhance bone formation [7,8,9,10,11,12,13,28,29,30,31,32,33]. For example, resveratrol treatments enhanced osteoblast viability and suppressed bone loss by altering *BMP2*, *Runx2* and *SIRT1* mRNA expression and reducing *RANKL*-stimulated bone loss [11,34,35,36]. Fruit extracts containing high levels of anthocyanins, as well as purified anthocyanins, decreased bone loss in animal models and increased osteoblast viability, suggesting that they have both antiresorptive and anabolic effects on bone tissues; however, the mechanisms have not been completely elucidated [7,8,9,10,11,12,13,37,38]. Previously, we reported that RES and POG treatment of *Sp7/osterix*:mCherry double-transgenic medaka enhanced osteoblastogenesis, and decreased Rankl-stimulated osteoclast formation and bone loss in *col10a1*:nlGFP/*rankl*:HSE:CFP triple transgenic medaka, suggesting that these two natural compounds have both anabolic and antiresorptive effects on bone [11]. Since our previous data suggested multiple mechanisms of action for these compounds, we established a global transcriptomic profile for cultured hFOB human osteoblasts treated with POG and RES to more fully understand the molecular and biological mechanisms of action. 

In view of the fact that mRNA and proteins are the primary molecules responsible for cell viability and function, the use of deep RNA sequencing and proteomics can significantly increase our understanding of the biological and molecular changes occurring in cells treated with small molecules. In our investigation, whole transcriptome profiling using mRNA-seq revealed that of 29,762 genes in cultured serum-starved hFOB osteoblasts treated with POG, exhibited 3177 DGEs (1481 upregulated and 1696 downregulated, FDR < 0.05), while treatment with RES resulted in 2288 DGEs (1068 upregulated and 1220 downregulated). As compared with negative controls, POG and RES had an overlap of only 18 DEGs (Log FC of <−1 to >1, FDR < 0.05), suggesting the potential for different mechanisms of action. In addition, IPA analysis revealed that POG altered gene expression in osteoblasts that overlapped with 125 canonical pathways, while RES altered genes in only 53 canonical pathways, indicating that POG has a more significant impact on cellular genomics. 

Removal of FBS from the medium (serum starvation, negative controls) reduced the proliferation of osteoblasts and induced apoptosis as compared with positive controls. Transcriptomic analysis of these osteoblasts revealed that, as compared with the positive controls, the negative controls exhibited significantly higher expression of genes that favored apoptosis. Of interest were the significant downregulation of *Bcl-2A1* (−2.5, FDR < 0.0001) and upregulation of *BIM* (FDR < 0.0001, *Bcl2L11* gene) *Bad* and *Bax* in negative control serum-starved osteoblasts. These genes encode for the B-cell lymphoma 2 (Bcl-2) proteins that regulate apoptosis and induce mitochondrial cytochrome c release, thereby stimulating apoptosome formation and caspase 9 activation, initiating intrinsic apoptosis [39,40]. *Bcl-2A1* has been reported to bind to *BIM*, *Bak* and *Bax*, three proapoptotic genes, and downregulate their expression [40,41]. As expected, *BIM*, *Bak* and *Bax* gene expression were upregulated in negative controls, indicative of apoptosis and supporting previous observations [40,41]. *BIM* expression activates *Bax/Bak*, thereby inducing apoptosis by mitochondrial release of cytochrome c and caspase (aspartate-specific cysteine protease activation) [41,42]. Confirming our previous work, removal of FBS from the media of hFOB osteoblasts induced apoptosis by altering gene expression for the Bcl-2 family of proteins to favor apoptosis [11]. Interestingly, RES or POG treatment (in low concentrations) of serum-starved osteoblasts led to improved viability and growth, as well as the downregulation of the canonical apoptosis pathway, similar to the positive controls. Whole-genome transcriptomic profiling of the same cells showed alterations in gene expression in the apoptosis canonical pathway, indicating that RES or POG treatments reduced apoptosis in serum-starved osteoblasts. As compared with negative controls, RES- and POG-treated serum-starved human osteoblasts exhibited a significant upregulation of *Bcl-2*, *Bcl-XL* and *Bcl-2A1* mRNA (FDR < 0.05), while *BIM* (*Bcl2L11* gene) and caspase 9 mRNA were significantly downregulated (FDR < 0.05). He et al. [43] previously reported that resveratrol reduced apoptosis in cultured mouse osteoblasts by increasing the expression of Bcl-2 proteins. However, alteration of *Bcl-2A1* mRNA expression by RES in osteoblasts has not been previously reported. Transcriptomic analysis also showed that RES significantly downregulated *BIM* mRNA expression, as well as caspases 3 and 9. These results support those of Tang et al. [44], who showed that *BIM* expression was downregulated in rat osteoblasts grown in a high-glucose medium and treated with RES. 

POG treatment of osteoblasts also improved cell growth and reduced apoptosis. Transcriptional analysis showed significant upregulation of *Bcl-2*, *Bcl-2A1* and *Bcl-XL* (FDR < 0.05), and significant downregulation of mRNA expression of *BIM* (FDR < 0.05). We have previously reported that POG treatment increased the Bax/Bcl-2 ratio in osteoblasts as determined by qPCR and reduce cell apoptosis [10,13], however, alterations in *BIM*, *Bcl-XL* and *Bcl2A1* gene expression have not been previously reported. Thus, both RES and POG in low concentrations appear to improve the cell culture milieu, similar to the addition of 10% FBS to the media, thereby improving osteoblast viability and transcriptionally altering gene expression associated with intrinsic apoptosis.

In addition to an increase in osteoblast viability, markers of osteoblast differentiation were also upregulated by RES and POG treatments. Osteoblast differentiation is a highly organized process and is regulated by the transcription factors, *Runx2*, *Dlx5* and *Sp7/osterix* [45,46,47,48]. POG treatments of serum-starved osteoblasts significantly upregulated the expression of all of these transcription factors (FDR < 0.05) as compared with negative controls. Shakibaei et al. [49] reported that RES pretreatment of cultured, nicotinamide-treated mesenchymal stem cells significantly increased osteoblast differentiation by increasing the expression of *Runx2* and decreasing the expression of *PPAR-γ* [49]. In our previous work, we reported that both POG and RES treatment of *Sp7/osterix*:mCherry Japanese medaka enhanced osteoblast differentiation, as well as upregulated the expression of *Sp7/osterix*, a zinc finger transcription factor [11]. Thus, the data presented in this work corroborates these previous investigations, and further supports the hypothesis that POG induces osteoblast differentiation by altering the expression of these critical transcription factors. Interestingly, POG, but not RES, significantly increased the expression of *Dlx5* (Distal-Less Homeobox 5, 5-fold, FDR < 0.05) mRNA in serum-starved osteoblasts. In mammalian bone, there are six Dlx transcription factors (Dlx1-6) that are important for osteogenesis [46,47,48]. Dlx5 is expressed in early bone development and controls the expression of many bone-related genes, thus playing a central role in osteogenesis [46,47,48]. Coimmunoprecipitation studies have reported a link between *Dlx5* and *Runx2* and have further shown that *Sp7/osterix* is a direct target of both *Dlx5* and *Runx2* [46]. Thus, our data indicate that POG likely exerts an anabolic effect by increasing *Dlx5*, which then targets *Runx2* and *Sp7/osterix*, resulting in osteoblast differentiation.

Osteoblasts are pivotal not only for bone-forming activities, but they are critical for maintaining bone homeostasis by altering the expression of molecules needed for osteoclast differentiation and function, including the receptor activator of NF-κβ ligand (Rankl) and osteoprotegerin (OPG) [50,51,52]. *Rankl* is expressed in osteoblasts and is released to bind to its receptor Rank on the osteoclast cell surface, which leads to the activation of osteoclastogenesis [50]. OPG is the receptor decoy for Rankl, and serves to prevent Rankl from binding to Rank, thereby inactivating osteoclasts [50]. Compounds that reduce the Rankl:OPG ratio can reduce osteoclastogenesis. Rankl, a transmembrane protein, is produced by osteoblasts and is cleaved to make the active soluble form by matrix metalloproteases (MMP3/7) and ADAM [50,51,52]. Interestingly, both estrogen and parathyroid hormone are known to reduce *Rankl* expression and increase OPG in osteoblasts, thereby increasing bone formation [51,52]. In this work, transcriptional analysis of RES- and POG-treated osteoblasts also showed a significant downregulation of *TNFSF11* (*Rankl*) but little effect on *OPG* (*TNFRSF11B*). qPCR analysis of RES- and POG-treated osteoblasts confirmed downregulation of *Rankl* mRNA expression. However, reduced *Rankl* expression alone would lead to a reduction in the Rankl:OPG ratio, ultimately reducing osteoclast differentiation and bone resorption. In RES-treated osteoblasts, both *MMP3* and *ADAM* were also significantly downregulated. Shakibaei et al. showed that high-density bone cultures treated with Rankl exhibited increased NF-κB activation, leading to the formation of tartrate-resistant acid phosphatase-positive multinucleated cells that resembled osteoclasts [53]. Pretreatment of this cell line with RES reduced the effects of Rankl by suppressing the activity of the enzyme IκBα kinase [53]. Ameen et al. reported that male rats treated with RES had reduced Rankl expression, and reduced age-dependent bone loss [54]. Furthermore, previously we reported that both POG and RES reduced Rankl-stimulated bone loss and osteoclast differentiation in triple transgenic medaka [11]. Thus, this work supports these previous investigations and further suggests that both *MMP3* and *ADAM* may be involved in the mechanism by which RES reduces *Rankl* expression.

Of note, downregulation of *Rankl* expression in POG-treated serum-starved osteoblasts was associated with downregulation of the hypoxia-inducible factor (HIF-1α) canonical pathway. Hypoxia, a reduction in blood oxygen levels, leads to reduced ATP production in the mitochondria by oxidative phosphorylation, and increased oxidative stress and toxic reactive oxygen species accumulation, which are correlated with bone resorption [55,56,57]. Signaling in this pathway is mediated by hypoxia-inducible factors (HIFs), whose expression remains low under normal oxygen levels but is increased during hypoxia [58,59]. Hypoxia and HIF-1α have been associated with numerous metabolic bone disorders, including osteoporosis, osteonecrosis and disorders that impact osteoclast differentiation [58,59,60,61]. Hypoxic conditions have been reported to reduce osteoblast activity and differentiation through reduced *Runx2* expression [62]. In addition, increased HIF-1α/RANKL/Notch1 signaling stimulated macrophage differentiation into osteoclasts [63]. Thus, hypoxia reduces the activities of osteoblasts, and increases the activities of osteoclasts, which results in dysregulated bone homeostasis, reduced BMD and microarchitecture, thereby increasing bone fracture risk [55,56,57,58]. In this work, POG significantly downregulated the hypoxia canonical pathway, including the expression of *HIF-1α*, *HIF-3α* and *HIF-1α-AS2* (FDR < 0.05), while *HIF1AN* (a HIF1α inhibitor) was significantly upregulated (FDR < 0.05), indicating that this compound affects several signaling molecules within the HIF pathway. In addition, both POG and RES increased ATP levels in cultured serum-starved osteoblasts (Supplemental Figure S1). Downregulation of *HIF-1α/Rankl/Notch1* signaling in serum-starved osteoblasts provides further insight into the multiple canonical pathways that are associated with the anabolic activities of POG, as well as its antiresorptive effects.

Finally, numerous cytokines and networks have been identified and reported to impact osteoblast and osteoclast function, as well as the formation and resorption of bone [64,65]. Significant changes in specific cytokine levels appear to play a critical role in metabolic bone disorders, including osteoporosis [65]. The interleukins (IL)-6, IL-17, IL-18, and other proinflammatory cytokines have been reported to enhance Rankl-induced osteoclast differentiation [64,65]. Interestingly, RES-treated serum-starved osteoblasts showed a significant (FDR < 0.05) upregulation of interleukin-18 mRNA and signaling (IL-18, Supplemental Figure S2). Interleukin-18 is primarily synthesized in osteoblasts, macrophages, and Kupffer cells. While its role in osteoclastogenesis is controversial, IL-18 has been reported to increase the activities of TNF-α and Fas/FasL and osteoclast apoptosis [66,67]. Interleukin-18 expression was reported to be upregulated in rat osteoblasts treated with parathyroid hormone [67]. Its role in osteoclastogenesis is thought to be similar to that of IL-1 and TNF-α; however, there are reports that contradict this hypothesis [68]. While POG treatments of osteoblasts did not affect IL-18, POG treatment reduced IL-6 mRNA expression (−3-fold, *p* < 0.05). Interleukin-6 has been shown to increase the RANKL:OPG ratio by increasing Rankl levels and reducing OPG, leading to excessive osteoclast activation and bone loss [69]. However, again, this is speculative, as there appears to be some controversy concerning IL-6 and its role in osteoclastogenesis [69].

## 5. Conclusions

Treatment of serum-starved hFOB human osteoblasts with low concentrations of POG or RES led to improved osteoblast viability and reduced apoptosis. Whole-genome transcriptomic profiling of the osteoblasts showed that both treatments altered DGE that overlapped with the apoptosis canonical pathway, favoring cell growth, further supporting these observations. POG treatment increased *DLX5* mRNA expression, a transcription factor associated with osteoblast differentiation and upregulated *Sp7/osterix* and *Runx2* mRNA expression. Both RES and POG significantly downregulated *Rankl* mRNA expression, likely leading to a reduction in the Rankl:OPA ratio that would suppress osteoclastogenesis, supporting the results of our previously published in vivo studies in transgenic medaka. Interestingly, POG-treatment of serum-starved osteoblasts also reduced HIF1α canonical pathway signaling, and both POG and RES altered the expression of proinflammatory interleukins, which are thought to be involved in osteoclastogenesis. These data suggest that these naturally occurring compounds have both anabolic effects (improved osteoblast viability and function), and anti-resorptive activities by significantly downregulating Rankl expression, as well as altering signaling in multiple canonical pathways and biological networks. These data support their possible use for the treatment of metabolic bone diseases, including osteoporosis.

## Figures and Tables

**Table 1:** (**A**) The top 10 upregulated DGEs are presented for POG-treated serum-starved osteoblasts. (**B**) The top 10 downregulated DGEs are presented for POG-treated serum-starved osteoblasts. (**C**) The top 10 upregulated DGEs are presented for RES-treated serum-starved osteoblasts. (**D**) The top 10 downregulated DGEs are presented for RES-treated serum-starved osteoblasts. Tables were generated using a q < 0.05 and FC of <−1 or >1.

**A**		**B**	
**Geneid**	**Gene name**	**Geneid**	**Gene name**
**ENSG00000120915**	EPHX2	ENSG00000282608	ADORA3
**ENSG00000267677**	RP11-27G24.1	ENSG00000213761	MT1P1
**ENSG00000273313**	RBAKDN	ENSG00000283045	RP11-764D10.2
**ENSG00000259215**	RP11-253M7.4	ENSG00000227517	LINC01483
**ENSG00000251600**	RP11-673E1.1	ENSG00000169442	CD52
**ENSG00000268750**	CTD-2583A14.10	ENSG00000274421	RP11-386J22.3
**ENSG00000159496**	RGL4	ENSG00000280639	LINC02204
**ENSG00000110328**	GALNT18	ENSG00000267938	EIF1P6
**ENSG00000260331**	RP11-111J6.2	ENSG00000198844	ARHGEF15
**ENSG00000233087**	WTH3DI	ENSG00000261783	RP11-252K23.2
**C**		**D**	
**Geneid**	**Gene name**	**Geneid**	**Gene name**
**ENSG00000196415**	PRTN3	ENSG00000159239	C2orf81
**ENSG00000120915**	EPHX2	ENSG00000118017	A4GNT
**ENSG00000227076**	RP11-4C20.4	ENSG00000241112	RPL29P14
**ENSG00000183484**	GPR132	ENSG00000109424	UCP1
**ENSG00000173930**	SLCO4C1	ENSG00000261832	RP11-435I10.4
**ENSG00000260947**	RP11-384P7.7	ENSG00000144868	TMEM108
**ENSG00000149633**	KIAA1755	ENSG00000236670	KRT18P5
**ENSG00000137843**	PAK6	ENSG00000235651	AC064850.4
**ENSG00000144488**	ESPNL	ENSG00000205502	C2CD4B
**ENSG00000183807**	FAM162B	ENSG00000198535	C2CD4A

## Data Availability

The mRNA-seq data presented in this manuscript are publicly available, have been deposited in NCBI’s Gene Expression Omnibus [24,25], and are accessible through GEO series accession number GSE200684, (https://www.ncbi.nlm.nih.gov/geo/query/acc.cgi?acc=GSE200684 accessed on 15 June 2023).

## Supplementary Materials

The following supporting information can be downloaded at: https://www.mdpi.com/article/10.3390/nu15143233/s1, Figure S1: ATP levels in resveratrol and peonidin-3-*O*-glucoside treated osteoblasts; Figure S2: Impact of resveratrol on IL-18 expression and signaling in osteoblasts. Figure S3A–E. qPCR analysis of Rankl, Dlx5, Sp7/osterix, and Runx2 mRNA expression in resveratrol and peonidin-3-*O*-glucoside treated osteoblasts.
