# Supplementary material for: Peonidin-3-O-glucoside and Resveratrol Increase the Viability of Cultured Human hFOB Osteoblasts and Alter the Expression of Genes Associated with Apoptosis, Osteoblast Differentiation and Osteoclastogenesis
Source: Nutrients. 2023 Jul 21;15(14):3233. doi: 10.3390/nu15143233 (PMC10383121; doi:10.3390/nu15143233)
Supplement: Supplementary file 1 [file nutrients-15-03233-s001.zip › nutrients-2467431-supplementary.pdf]

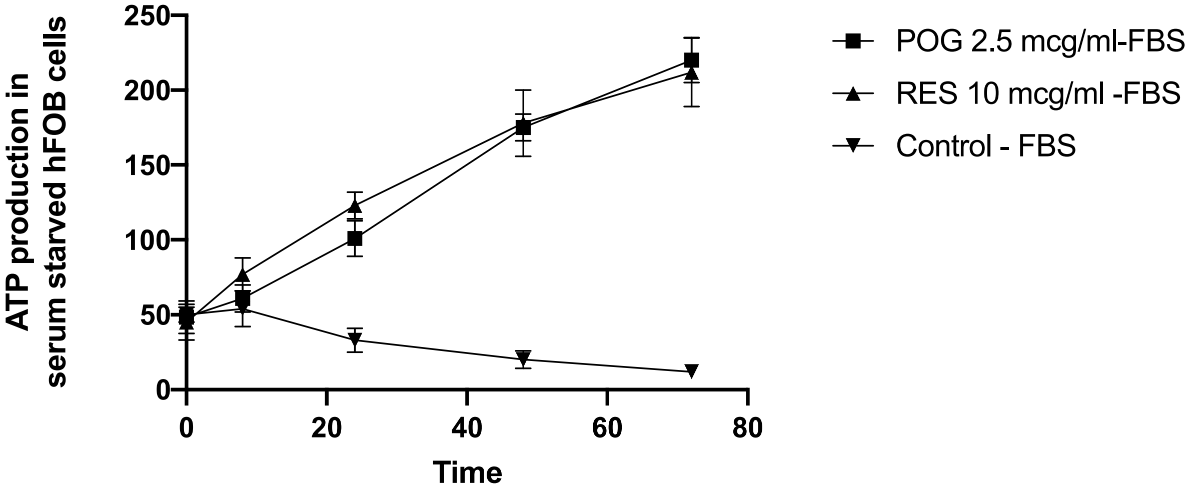


**Supplemental Figure S1.** ATP concentrations in serum starved osteoblasts treated with RES (10 mcg/ml) or POG (2.5 mcg/ml) for 72 hours. ATP concentrations in the negative controls (serum starved osteoblasts treated only with vehicle solvent, -FBS) remained low over the treatment period. Osteoblasts treated with POG or RES showed an increase in ATP concentrations starting at 4 hours and continuing for 72 hours. Experiments were performed in triplicate.

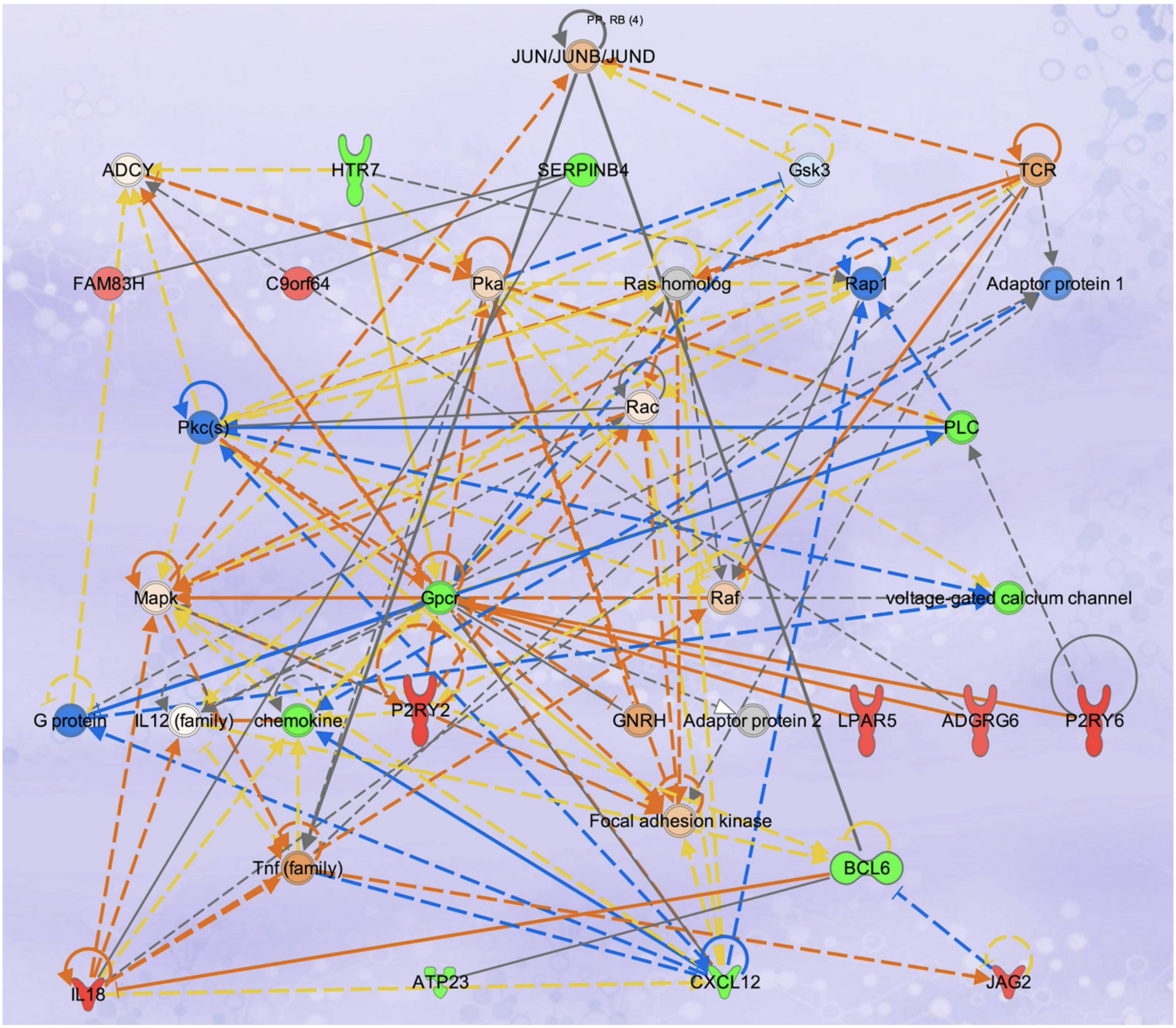


**Supplemental Figure S2.** Network analysis of the effects of resveratrol on serum starved cultured human osteoblasts on IL-18 mRNA expression and gene expression within this signaling pathway. Generation of this plot in Ingenuity Pathway Analysis (IPA) was performed using an FC of <-1 or >1 with a false discovery rate FDR <0.05. The analysis was based on the ratio of the number of DEGs in the dataset to the total number of reference genes in corresponding pathways in the IPA knowledge bases. The IPA analysis used the Fisher's exact test ($p < 0.05$) to determine the significant networks that correlated with the DEGs from the RNA-seq data. Red/pink colors indicate significant upregulation of genes, while green indicates significant downregulation of genes. The blue color indicates predicted downregulation of gene expression. Enrichment of the DEGs was determined using the Ensembl database.

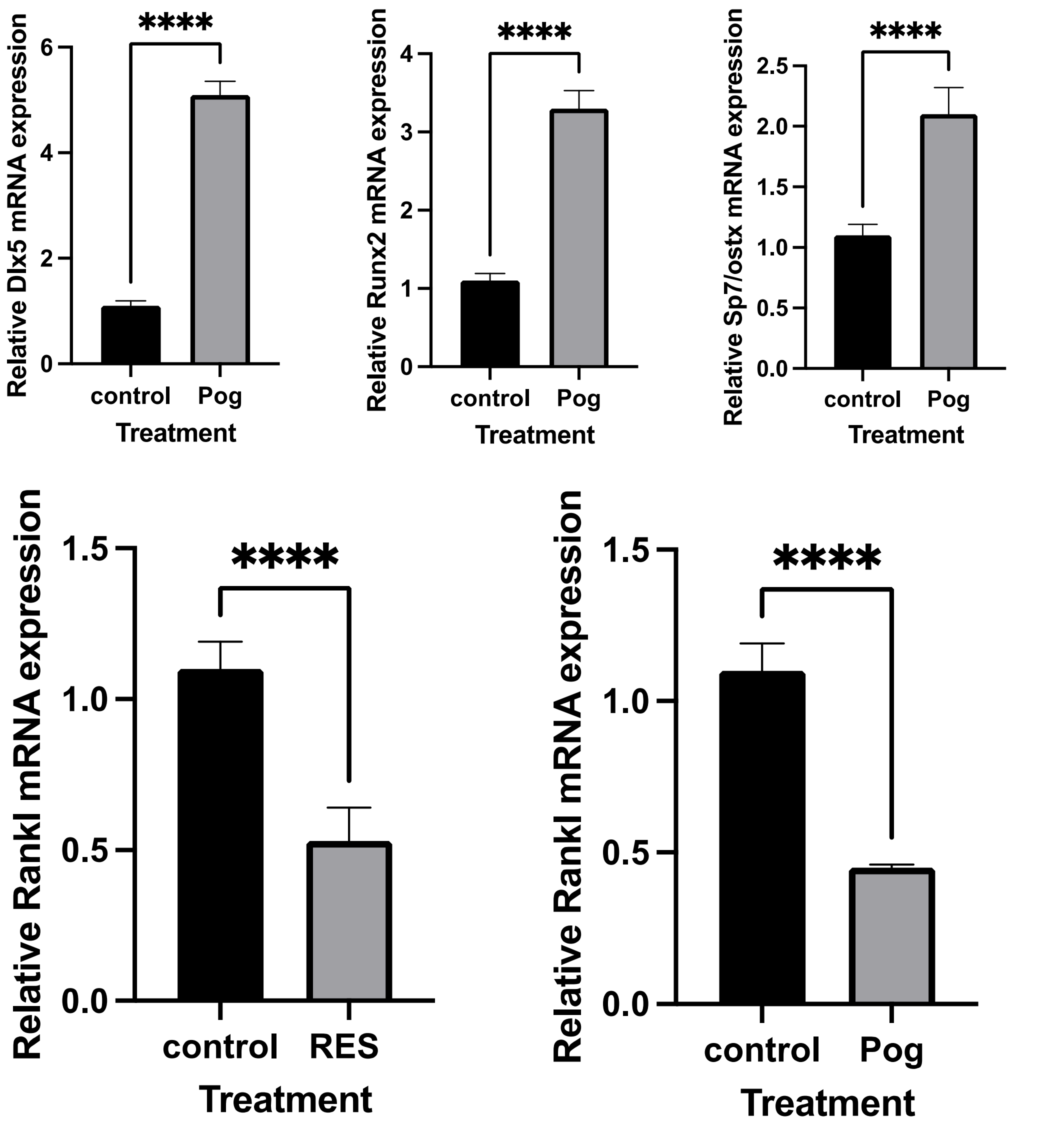


**Supplemental Figure S3A-E.** qPCR analysis of *Rankl, Dlx5, Sp7/osterix* and *Runx2* mRNA expression in resveratrol and peonidin-3-*O*-glucoside treated osteoblasts. Controls were serum starved osteoblasts treated with 0.01% DMSO (vehicle solvent). **A**. Treatment of the serum starved osteoblasts with POG (1.0 µg/ml) increased *Dlx5* mRNA expression as compared with negative controls and increased cell viability. **B.** Treatment of the serum starved osteoblasts with POG (1.0 µg/ml) increased *Runx2* mRNA expression as compared with negative controls. **C.** Treatment of the serum starved osteoblasts with POG (1.0 µg/ml) increased *Sp7/osterix* mRNA expression as compared with negative controls. **D.** Treatment of the serum starved osteoblasts with RES (1.0 µg/ml) decreased *Rankl* mRNA expression. **E.** Treatment of the serum starved osteoblasts with POG (1.0 µg/ml) decreased *Rankl* mRNA expression as compared with negative control cells treated with DMSO 0.01% (vehicle solvent). Gene expression was quantified using endogenous control β-actin gene (primer as we have described in ref 11] and using the $^{\Delta\Delta}$CT

calculation. Statistics were performed using the unpaired T-test with Welch's correction using GraphPad 10.0 (San Diego, CA). Primers: Rankl
Forward: GCCTTTCAAGGAGCTGTGCAAAA, Backward: GAGCAAAAGGCTGAGCTTCAAGC; Dlx5 Forward:TACCCAGCCAAAGCTTATGCCG, Backward: GCCATTCACCATTCTCACCTCG; Runx2
Forward: CCCAGTATGAGAGTAGGTGTCC, Backward: GGGTAAGACTGGTCATAGGACC; Sp7/Osterix
Forward: TTCTGCGGCAAGAGGTTCACTC, Backward: GTGTTTGCTCAGGTGGTCGCTT (Origene, Rockville, MD).
